# miRNome profiling in Duchenne muscular dystrophy; identification of asymptomatic and manifesting female carriers

**DOI:** 10.1042/BSR20211325

**Published:** 2021-09-17

**Authors:** Nahla O. Mousa, Ahmed A. Sayed, Nagia Fahmy, Mariam G. Elzayat, Usama Bakry, Ahmed Abdellatif, Waheed K. Zahra, Ahmed Osman

**Affiliations:** 1 Biotechnology Department, Basic and Applied Sciences Institute, Egypt-Japan University of Science and Technology, Borg Al Arab 21934, Egypt; 2 Biotechnology Department, Faculty of Science, Cairo University, Giza 12613, Egypt; 3 Genomics Program, Children’s Cancer Hospital Egypt 57357, Cairo, Egypt; 4 Biochemistry Department, Faculty of Science, Ain Shams University, Cairo 11566, Egypt; 5 Neuromuscular Unit, Neuropsychiatry Department, Faculty of Medicine Ain Shams University, Cairo 11566, Egypt; 6 Biology Department, and Biotechnology program, School of Sciences and Engineering, The American University in Cairo, School of Sciences and Engineering, Cairo 11835, Egypt; 7 Mathematics Department, Basic and Applied Sciences Institute, Egypt-Japan University of Science and Technology, Borg Al Arab 21934, Egypt; 8 Physics and Engineering Mathematics Department,Faculty of Engineering, Tanta University, Tanta 31733, Egypt

**Keywords:** Carriers, Duchenne muscular dystrophy, microRNA, Next generation sequencing

## Abstract

Duchenne muscular dystrophy (DMD) is a fatal neuromuscular disorder that occurs due to inactivating mutations in DMD gene, leading to muscular dystrophy. Prediction of pathological complications of DMD and the identification of female carriers are important research points that aim to reduce disease burden. Herein, we describe a case of a late DMD patient and his immediate female family members, who all carry same DMD mutation and exhibited varied degrees of symptoms. In our study, we sequenced the whole miRNome in leukocytes and plasma of the family members and results were validated using real-time PCR. Our results highlighted the role of miR-409-3p, miR-424-5p, miR-144-3p as microRNAs that show correlation with the extent of severity of muscular weakness and can be used for detection of asymptomatic carriers. Cellular and circulating levels of miR-494-3p had shown significant increase in symptomatic carriers, which may indicate significant roles played by this miRNA in the onset of muscular weakness. Interestingly, circulating levels of miR-206 and miR-410-3p were significantly increased only in the severely symptomatic carrier. In conclusion, our study highlighted several miRNA species, which could be used in predicting the onset of muscle and/or neurological complications in DMD carriers.

## Introduction

Duchenne muscular dystrophy (DMD; OMIM reference 310200) is a neuromuscular disorder characterized by a rapid deterioration of the skeletal musculature, in particular the proximal muscles [1]. DMD is caused by mutations in the largest gene in the human genome known as DMD gene (Xp21.2), which encodes dystrophin protein, resulting in production of a c-terminal truncated protein product [2]. Dystrophin is primarily found in muscles and connects the membrane of the muscle fiber to the sarcolemma via linking actin filaments to dystrophin-associated glycoprotein complex (DGC) located in the inner side of sarcolemma [3]. Carboxy-terminal truncation of dystrophin causes membrane destabilization and increased extracellular Ca^2+^ influx into myocytes, which activates apoptotic mechanism leading to muscle atrophy and fibrosis due to the repeated muscle degeneration–regeneration cycles [4]. In advanced stages, severe cardiac and pulmonary involvement are recognized and considered the leading reason for mortality [5]. The disease affects mainly male births due to its X-linked recessive nature. On the other hand, female carriers are mostly asymptomatic, but some carriers may show varied degree of disease symptoms and hence are called manifesting carriers [6].

Diagnosis of DMD depends mainly on the clinical presentation of the patient and some non-specific measures like the high blood levels of creatine kinase (CK), muscle MRI and presence of cardiac complications [7]. When DMD is suspected, genetic testing is carried out to identify the causative DMD mutation(s). To identify large deletions/duplications, multiplex ligation-dependent probe amplification (MLPA) [8] is carried out as an initial diagnostic test [9], however, it can only detect genetic aberration in ∼70% of the patients [10]. When MLPA results come negative, analysis of small and micro deletion/duplication and point mutations is performed using next-generation sequencing (NGS) [11]. Nonetheless, the later technique is expensive and sometimes deep intronic mutations and small insertions may escape detection by this method [12]. In such condition, clinicians may resort to detecting protein aberrations in muscle biopsies by Western blotting and immunohistochemical analysis [13]. Besides being invasive, such techniques are time-consuming and hence, further studies are carried out for searching for reliable alternative approaches. In addition, inheritance of a defective maternal allele of the DMD gene accounts for approximately two-thirds of disease onset, and thus detection of DMD mutation(s) in female carriers could present a challenging, yet important task to reduce disease burden [14].

MicroRNAs are intracellular post-transcriptional regulators that play a crucial role in the organization of gene expression inside the tissues. Also, microRNA can be released from one cell to exert its effect on a distant cell and thus some microRNA species are found to be circulating in various body fluids [15]. The levels of circulating miRNAs could be changed in various pathological conditions either due to abnormal intracellular expression or their release from the damaged tissue [16]. Consequently, many studies attempted to establish some miRNA species as diagnostic/prognostic biomarkers that can detect the onset and predict disease progression as well as evaluate responses to therapeutic interventions [17]. Previous reports highlighted the significance of the dysregulation of the levels of target miRNA species in muscle biopsies of DMD patients and in animal models (mdx mice or golden retriever muscular dystrophy (GRMD) dog model) [18,19]. However, very few studies were carried out to assess the microRNA profiles in the sera of DMD patients [20–22], and to our knowledge, no studies were performed to screen the dysregulation in miRNA profiles in female carriers using high throughput techniques. Detecting the roles of miRNAs in disease etiology and investigating the levels of microRNAs is usually done using real-time PCR and microarray. However, applying NGS approach for analyzing the levels of the microRNAs in the plasma samples of DMD patients will give a clearer picture about the miRNA species whose levels are significantly altered, and thus, can be correlated with and shed light on to the clinical presentation of the disease.

Among the various species of microRNAs, some were found to be solely expressed in skeletal muscles including miR-206, miR-486, and miR-499 [23]. In a study by Li et al., the authors reported that the serum levels of some microRNAs could have diagnostic and prognostic role in DMD patients [24]. Anaya-Segura et al. also concluded that up-regulated serum levels of miR-206 could be used to detect DMD female carriers [25]. Our previous findings also showed that plasma miR-499 was significantly up-regulated in DMD patients and had the ability to identify female carriers [21].

In the present study, we employed a massively parallel sequencing approach to carry out miRNA profiling in a DMD patient, his mother and two sisters who were found to carry DMD mutation by MLPA in an attempt to search for the differentially expressed microRNAs that can act as non-invasive biomarkers. In addition, identifying the dysregulated microRNAs will provide insights about the pathophysiology of the disease and the phenotypic features of DMD boys and female carriers.

## Methods

### Human subjects

The present study represents a family report in which the members of one family (DMD patient, his mother, and two sisters) were enrolled and diagnosed using MLPA and shown to have aberration in the dystrophin gene (deletion of exons 46–50; the mother (DF2/NA; 45 years, asymptomatic carrier with normal CK levels), the elder sister (DF3/HB; 27 years, symptomatic carrier who had cramps of lower limbs and had high levels of CK in blood), the younger sister (DF4/HA; 12 years, with borderline IQ, muscle cramps of lower limbs and high CK in blood), and a son (DF1/WA, 19 years). In addition, two control subjects were enrolled in the present study, a female with comparable age as of the older sisters in addition to a control male with similar age to that of the DMD boy.

### Sample collection and RNA isolation

Peripheral blood samples were collected from the enrolled subjects in EDTA-containing vacutainers. Plasma and blood cells were isolated from the whole blood using density gradient centrifugation. MicroRNA extraction was conducted using miRNeasy Mini kit (Qiagen, Germany; Cat. No.: 217084) following the manufacturer’s recommendations and the concentration of RNA samples was determined using the Qubit RNA HS Assay Kit (Invitrogen, U.S.A.; Cat. No.: Q32852).

### miRNA library preparation

RNA libraries were generated using NEXTFLEX® Small RNA-Seq Kit (PerkinElmer, U.S.A.; Cat. No.: NOVA-5132-05). For each library, 400 ng purified RNA was used as an input for library preparation according to the manufacturer’s instructions. Ligated libraries were reverse transcribed and amplified with a unique barcode primer for each one. DNA fragments ∼150 bp (miRNA sequences plus 3′ and 5′ adaptors) were determined using 10% TBE-PAGE gel then retrieved in a 300-µl elution buffer for purification. The size distribution of the pooled library was checked using the Bioanalyzer DNA assay (Agilent, U.S.A.; Cat. No.: 5067-1504) and the concentration was assessed using the Qubit dsDNA HS Assay (Thermo Fisher Scientific, U.S.A.; Cat. No.: Q33230). The final pooled library was sequenced for ∼2400 miRNAs using Illumina MiSeq system (Illumina, Inc., U.S.A.).

### Quality control, alignment, and quantification

**Figure 1:**
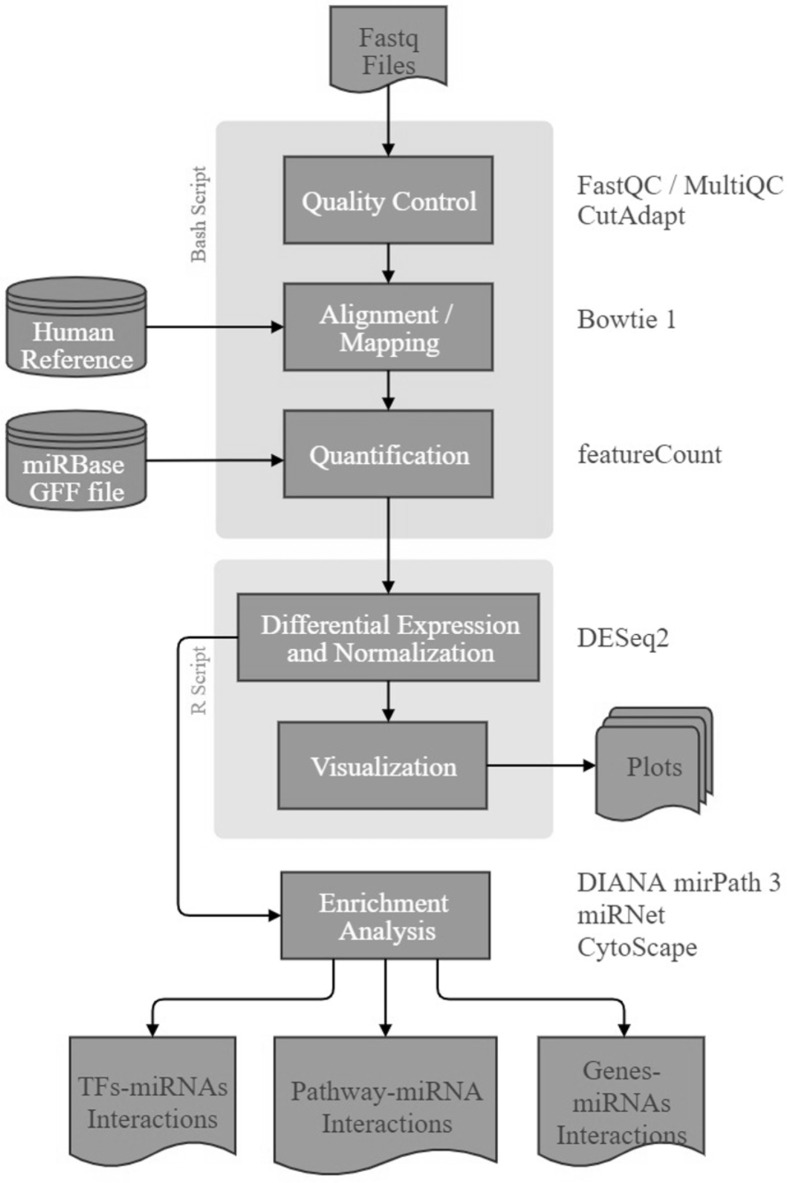
Bioinformatics workflow The workflow shows the bioinformatics pipeline used to analyze the miRNA data generated from Illumina MiSeqDx. The pipeline consists of six components: (1) Quality control, (2) Alignment, (3) Quantification, (4) Differential expression and normalization, (5) Visualization, and (6) Pathway analysis.

The quality control of the generated single reads for each sample was performed using FastQC, MultiQC [26], and CutAdapt and high quality (mean per-base-sequence-quality ≥ 30) filtered reads were then aligned against miRBase database (http://www.mirbase.org/) [27] using Bowtie 2 aligner. After that, the quantification step was performed using Samtools [28,29] to facilitate the differential expression and normalization step (Figure 1).

### Differential expression, normalization, visualization, and pathway analysis

The results of the quantification step were imported to Rstudio for further analysis [30]. In order to normalize miRNA counts and detect the differentially expressed miRNAs (DEMs), a Bioconductor DESeq2 R package was used [31]. DEMs were detected based on the adjusted *P*-value <0.1 and Log2 Fold Change > 2. The results of the analysis were visualized in volcano plots, heatmaps, and lollipop charts using ggplot2 R package [32]. Finally, pathway and disease transcription factors analysis were performed using DIANA mirPath v3.0 (http://snf-515788.vm.okeanos.grnet.gr/) [33] and TFmiR2 (http://service.bioinformatik.uni-saarland.de/tfmir2/) [34] respectively. All networks were visualized using Cytoscape [35] (Figure 1).

### Quantitative real-time PCR

In order to validate the miRNA sequencing results, quantitative RT-PCR analysis was carried out using StepOne™ Real-Time PCR System (Applied Biosystems). The miRNA-specific primers were ordered from Eurofins (Luxemborg). MicroRNA was extracted using miRNeasy kit (Qiagen, Germany; Cat. No.: 217084) and reverse transcribed using miScript II RT kit (Qiagen, Germany; Cat. No.: 218160) and qPCR performed using miScript SYBR green kit (Qiagen, Germany; Cat. No: 218073) according to the manufacturers instructions. Spike in Control (Qiagnen, Germany, Cat. No: 219610) was added to the samples before the extraction process to serve as a normalizer in the PCR amplification. The primer sequences used for miRNA quantification in real-time PCR are presented in Supplementary Table S1. All reactions were done in triplicates and no template controls in addition to negative RT reactions were included in the performed runs. Thermal cycling conditions were 15 min at 95°C, followed by 40 cycles at 95°C for 15 s and 55°C for 30 s and 70°C for 30 s. Melting curve analysis was included to assure the specificity of the amplification process. Results were analyzed using the comparative 2^−ΔΔ*C*_t_^ Livak method. Statistical analysis was carried out using a two-tailed *t* test.

## Results

### Clinical presentation and motor assessment of the DMD subject

**Figure 2:**
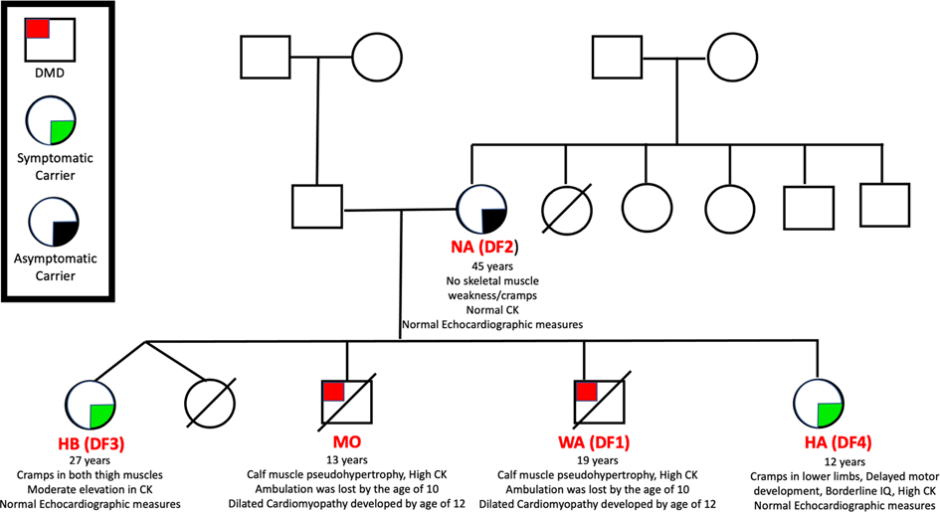
The family pedigree Proband (WA/DF1) is the male patient, the elder sister (HB/DF3), the younger sister (HA/DF4), and the mother (NA/DF2).

Thorough clinical examination was carried out for all family members (family pedigree is presented in Figure 2 and the clinical summary of the participants are presented in Table 1). The proband (WA) was a 19-year-old male patient with non-consanguineous parents. He had average motor, mental and speech development. When he was 5 years old, the family started to notice his slowness in running and frequent falling. Since that time, there was progressive proximal weakness of both lower limbs followed by trunk muscle weakness and proximal weakness of both upper limbs. On examination, he was found to have the phenotype of DMD with bilateral calf muscle pseudohypertrophy, positive Gower’s sign, and weakness of gluteal, thigh, gastrocnemius, paraspinal, deltoid and biceps brachii muscles. All deep tendon reflexes were diminished except intact ankle reflexes. The patient lost ambulation at the age of 10 years. His total CK level was in thousands at the time of the diagnosis of his condition, followed by a gradual decline until loss of ambulation. He also developed dilated cardiomyopathy at the age of 12 years and his ejection fraction started to decrease gradually. The patient was deceased at the age of 19.

**Table 1:** Baseline characteristics and clinical summary of DMD patient and the female carriers

Subject	Age at diagnosis	CK levels, U/l	Exon-Deletion pattern (MLPA)	Clinical diagnosis	Clinical information
Patient (DF1)	5 years	9432	Hemizygous pathogenic deletion encompassing exons 46–50	DMD	- Average motor, mental, and speech development bilateral calf muscle pseudohypertrophy, positive Gower’s sign, and weakness of gluteal, thigh, gastrocnemius, paraspinal, deltoid and biceps brachii muscles -Ambulation loss at age of 10 -Dilated cardiomyopathy at age of 12
Mother (DF2)	-	Normal	Heterozygous pathogenic deletion encompassing exons 46–50	DMD carrier	- Clinically free/asymptomatic
Elder sister (DF3)	20 years	500	Heterozygous pathogenic deletion encompassing exons 46–50	DMD carrier	-Pains and cramps in both the thighs -Bilateral calf pseudohypertrophy
Younger sister (DF4)	2 years	3392	Heterozygous pathogenic deletion encompassing exons 46–50	DMD carrier	- Delayed motor development - Poor scholastic achievement/borderline IQ - Outbursts of anger and abnormal behavior - No calf pseudohypertrophy - Intact deep tendon reflexes

The patient had an elder brother (MO) with a similar condition who lost ambulation at the age of 10 years, developed dilated cardiomyopathy at the age of 12 years, and suddenly died at the age of 13.5 years.

The elder sister (HB) is 27 years old and had a female twin who died immediately after birth. She had normal motor, mental, and speech development. She suffered no complaints until at the age of 20, she started to have pains and cramps in both thighs during walking. On examination, the motor power of both lower limbs, trunk, and upper limbs was average with intact deep tendon reflexes, but she had bilateral calf pseudohypertrophy. Her total CK level was mildly elevated; 500 U/l and her echocardiography showed average parameters.

The younger sister (HA) is 12 years old. She had delayed motor development as she sited at the age of 9 months and could walk alone at the age of 2 years. She had poor scholastic achievement and her IQ was classified as borderline. She was usually found as easily provoked with outbursts of anger and abnormal behavior. At the age of 6 years, she started to experience cramps of both lower limbs during walking and after physical exercises. On examination, she had no weakness of upper limbs, trunk and lower limbs; she had no calf pseudohypertrophy and intact deep tendon reflexes. Her total CK level was found to be high, 3392 U/l and her echocardiography showed average parameters. Her MRI brain was average, and her electroencephalogram (EEG) did not show any changes.

The patient’s mother (NA) is 45 years old, of non-consanguineous parents. She had two brothers and three sisters, one of whom died, who are free of any neurological abnormalities. The mother married at age of 17, gave birth to four children, of whom two were the deceased DMD boys. She did not experience any cramps or weaknesses and had an average neurological and neuromuscular examination. Her total CK level was within normal levels and she had average echocardiography parameters.

### Potential miRNA candidates for DMD diagnosis and carrier detection

In our attempt to identify dysregulated microRNAs in DMD, Illumina-based MiSeq massive parallel sequencing was utilized to identify differential patterns of circulating miRNAs in the plasma and the leukocytes in DMD patient and female carriers relative to control samples.

**Figure 3:**
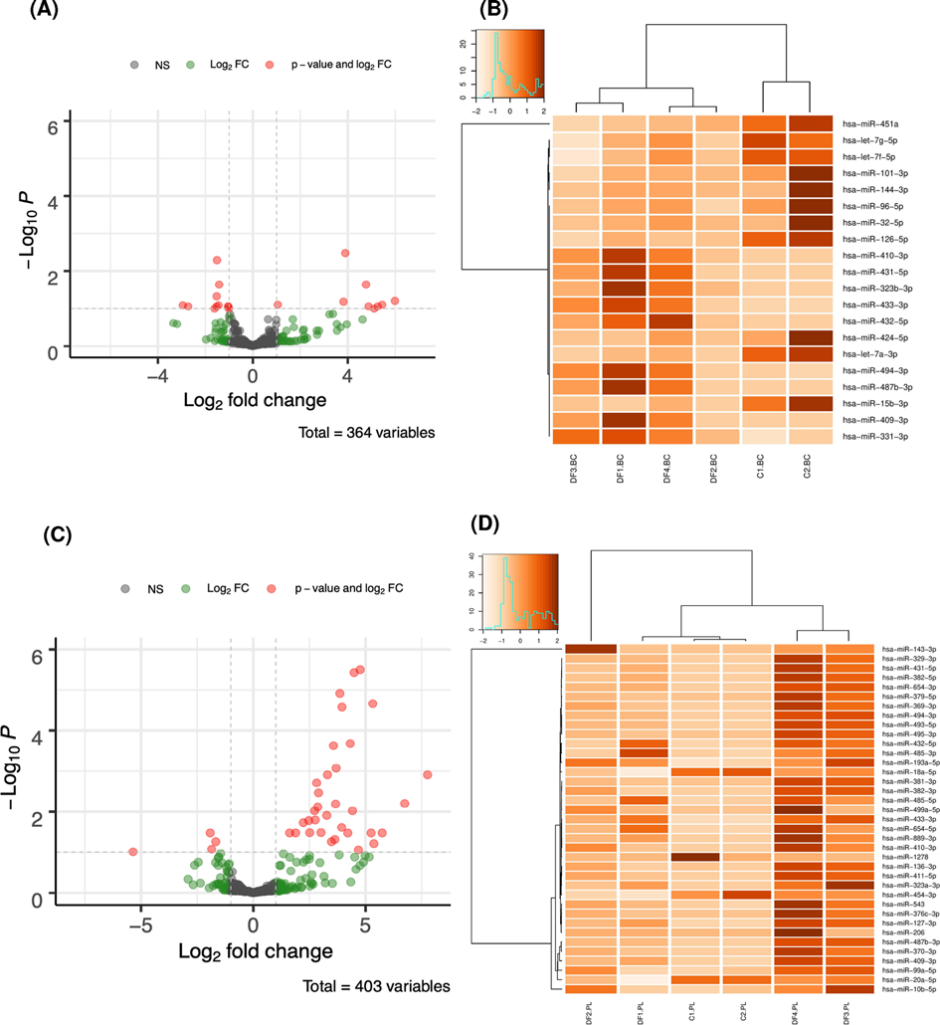
Volcano plots and heat maps (**A**) Volcano plot indicating the dysregulated miRNA species in the leukocytes of DMD subjects compared with the control subjects. Statistically significant species are represented as Red dots and Green dots. (**B**) Heat map with the scale bar (z-score) representing the significantly regulated miRNAs in the leukocytes of DMD patient and the female carrier detected through miRNA profiling. (**C**) Volcano plot indicating the dysregulated miRNA species in the plasma of DMD subjects compared with the control subjects. (**D**) Heat map with the scale bar (z-score) representing the significantly regulated miRNAs in the plasma of the enrolled DMD patient and the female carriers. **C1-PL:** male control, **C2-PL:** female control, **DF1-PL:** DMD boy, **DF2-PL**: mother of DMD boy, **DF3-PL:** elder sister (manifesting carrier), **DF4-PL:** younger sister (manifesting carrier).

In each run, six samples were tested, two control samples, one DMD patient, and three family members confirmed female carriers: one asymptomatic female carrier, one mildly symptomatic female carrier, and one symptomatic female carrier with neurological involvement. The read count per sample was ∼1204011 and 1416006 in the leukocytes and the plasma, respectively, after trimming of adaptors and excluding reads with low quality. DEMs in the leukocytes and the plasma DMD patient and female carriers and control subjects were visualized as heatmaps and volcano plots with adjusted *P*-value cutoff is 0.1; the cutoff in vertical axis is −*log*10(0.1) = 1 and the cutoff of the |*log*2*FoldChange*| > 1 (Figure 3).

**Figure 4:**
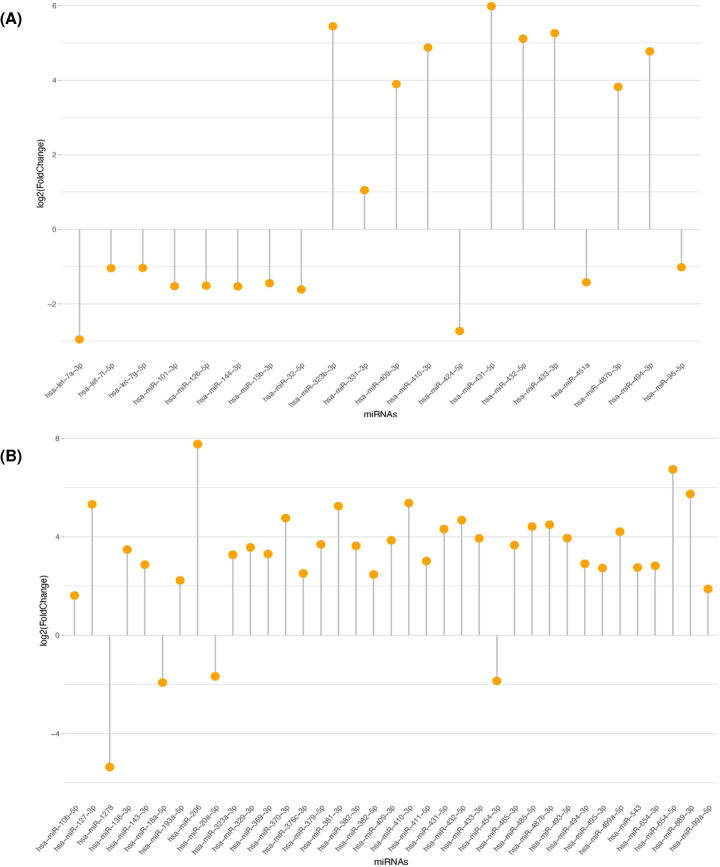
Lollipop plots displaying the dysregulated leukocyte miRNAs (**A**) Lollipop plots highlight 20 miRNA species dysregulated in the leukocytes of DMD boy and female carriers. (**B**) Lollipop plots that highlight 29 miRNA species dysregulated in the plasma of DMD boy and female carriers. The plots indicate the degree of fold change of selected microRNA species.

Our analysis revealed that 20 and 29 miRNA species were significantly altered in the leukocytes (Figure 4A) and the plasma (Figure 4B), respectively in the study test samples: the participated DMD patient and the three female carriers and can be utilized to diagnose DMD and carriers’ identification. Interestingly, the used controls an age-matched boy and a girl, showed no significant variations in their miRNA patterns, and thus, excluding presence of sex-related differences in dysregulated miRNA species. However, we opted to make pairwise comparisons between the DMD patient and the female carriers with the respective controls to eliminate the possibility of having any lapses in our data analysis or reached conclusion. The complete list of the microRNAs and their rankings are available in the Supplementary Tables S2–S9).

### MicroRNAs expression pattern in the DMD boy

We identified 48 statistically significant dysregulated miRNA species in the leukocytes of the DMD boy as compared with that of the corresponding control subject, of which 17 species were found to be down-regulated and 31 species were up-regulated (Supplementary Table S2).

On the other hand, a sum of 136 dysregulated miRNA species were found in the plasma of the DMD patient, 76 miRNAs of which were relatively low, while the remaining 60 species were significantly elevated as compared with that of the respective control (Supplementary Table S3). Comparative data analysis showed that some dysregulated miRNA species were detected as circulating in serum of the DMD patient and yet were modulated in his leukocytes. On the contrary, most of the dysregulated miRNAs were either found exclusively as circulating species in serum or uniquely expressed in leukocytes and this could be used for following up with disease progression when correlated with systemic complications.

### The expression profile of microRNAs in the female carriers

A major aim of our study was to screen for the dysregulated microRNAs in symptomatic and/or asymptomatic carriers to identify miRNA species and the target genes that play roles in the onset of symptoms or those that may carry diagnostic potential to identify symptom-free carriers, and thus, may help in pre-marriage genetic counseling.

From analyzing the levels of dysregulated miRNAs in the leukocytes and plasma of the DMD patient’s mother (asymptomatic carrier), it was found that 21 and 42 miRNA species, respectively, were altered as compared with the corresponding levels of the control female subject’s samples. Our data showed that 16 miRNAs were down-regulated and 5 species were up-regulated in leukocytes (Supplementary Table S4), while 22 miRNAs were down-regulated and 20 showed elevated levels (Supplementary Table S5).

**Figure 5:**
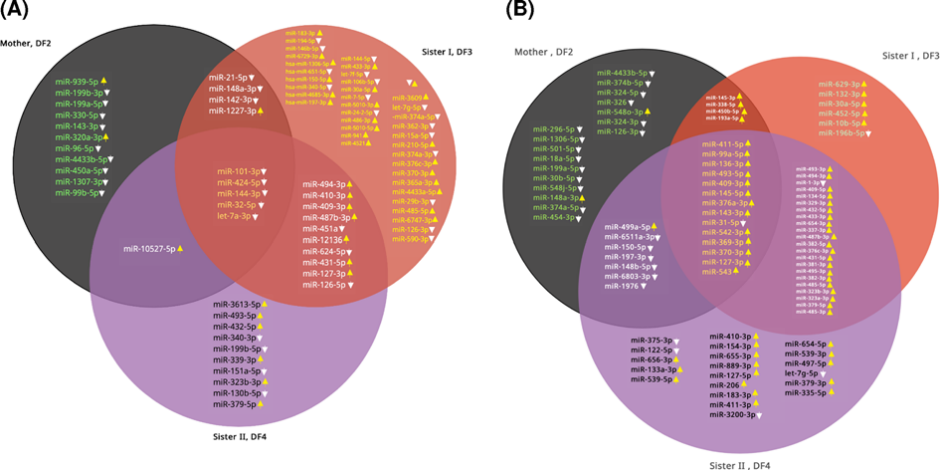
Common and unique miRNA species in female DMD carriers Venn diagram showing the unique and common miRNA species in the three female carriers. (**A**) Diagram represents the leukocyte-specific miRNAs and (**B**) the plasma-specific miRNAs.

Regarding symptomatic carriers, our data revealed that the miRNA profiles in the leukocytes and plasma of the older sister (HB) exhibited dysregulation in 57 and 46 miRNA species, respectively. Of those, 29 miRNAs were up-regulated in leukocytes and 43 species were also up-regulated as circulating levels in plasma, while 28 species showed relatively lower levels in leukocytes and only 3 species were low in plasma as compared with the levels of the respective control subject (Supplementary Tables S6 and S7). On the other hand, the younger symptomatic sister (HA) who showed significant neurological symptoms, exhibited modulation in miRNA profiles with 26 and 63 species showed variations of their levels in leukocytes and plasma, respectively when compared with that of the control female. Twelve miRNAs were down-regulated in leukocytes, and 11 species showed low circulating levels in plasma, while the rest of dysregulated miRNAs were up-regulated (Supplementary Tables S8 and S9). Figure 5 shows a Venn diagram, which displays the unique plasma miRNAs in each of the tested carriers as well as the common miRNA species between the two sisters and between each of them and their mother as well as the common ones in the three tested carriers.

Interestingly, we found 10 miRNA species that were dysregulated solely in the leukocytes of HA (DF4; the younger sister) (Supplementary Table S10), in addition to 20 other species that were also dysregulated in plasma (Supplementary Table S11). Such findings could be used to identify target genes and hence, explore the relationship between the occurrence of neurological/neuromuscular symptoms such as those existing in the tested carrier with the affected signaling pathways and consequently establish a set of biomarkers to be used as evidence of pronounced neurological involvement in the DMD cases. List of the ten most modulated microRNA species in the family members was included in Table 2.

**Table 2:** List of the ten most modulated microRNA species in the family members

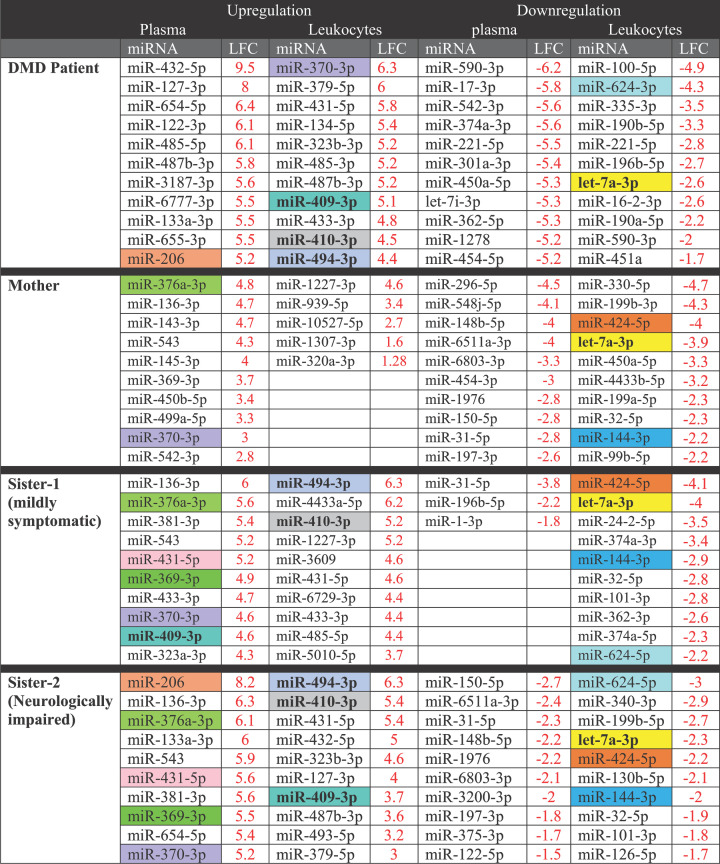

Highlighted cells represent common miRNA species. Abbreviation: LDC, log fold change value.

It is also worth mentioning that some miRNA species were dysregulated only in the two symptomatic sisters (HA and HB; DF3 and DF4) and these miRNAs would have a diagnostic potential to identify symptomatic female carriers and to detect life-threatening complications like deterioration of the skeletal muscle activity and the possibility of developing cardiomyopathy and as predictors of case severity.

**Figure 6:**
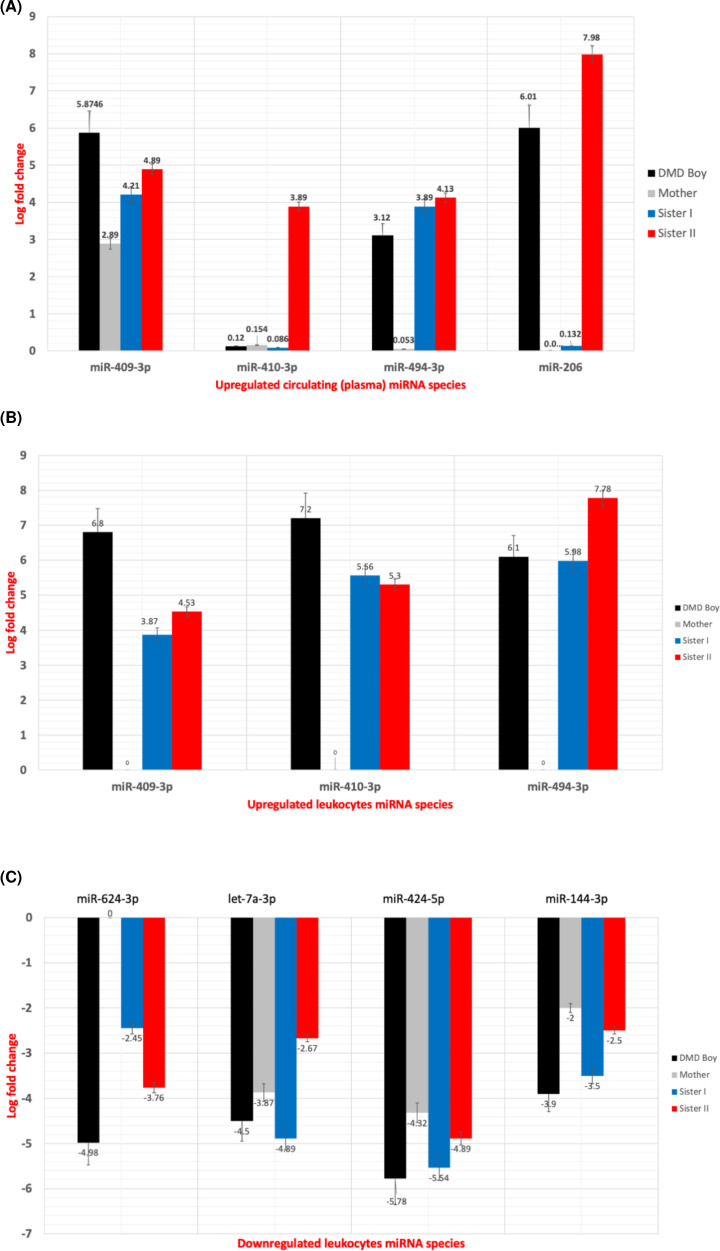
Real-time PCR validation results Graph representing the log-fold change in miRNA expression showing (**A**) the most dysregulated species in the plasma of the DMD patient and the female carriers, (**B**) the significantly up-regulated miRNAs in the leukocytes, and (**C**) the most down-regulated miRNAs in the leukocytes in all family members.

The generated NGS data were validated using real-time PCR (qPCR) to confirm the diagnostic potential of the selected miRNA species (Figure 6). Several miRNA species were quantified in the serum of the family members (Figure 6A); miR-409-3p was up-regulated in all family members in symptom-dependent manner and hence it could be used to detect both symptomatic and asymptomatic carriers. Also, the expression levels of miR-410-3p and miR-494-3p were elevated in the symptomatic carriers, however miR-206 was elevated in the DMD boy and the younger female carrier. Regarding the leukocyte–miRNAs, miR-409-3p, miR-410-3p and miR-494-3p were highly up-regulated in the DMD boy and the symptomatic carriers (Figure 6B), however, miR-624-3p was down-regulated (Figure 6C). Interestingly, let-7a-3p, miR-424-5p and miR-144-3p were significantly down-regulated in the family members (Figure 6C) in a pattern dependent on the muscle weakness.

### miRNA–mRNA target interactions

**Figure 7:**
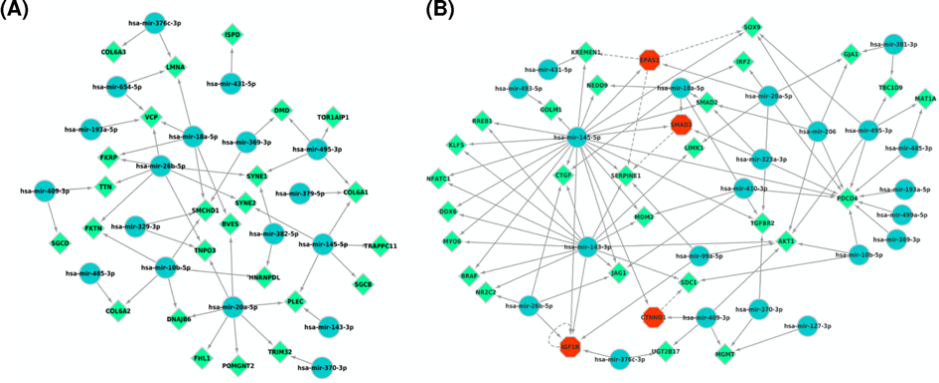
miRNA interactions (**A**) Network of miRNA–mRNA interactions identified by DIANA mirPath v3.0. The dysregulated microRNAs identified in the serum of the patients and female carriers are regulating a panel of protein-encoding genes like DMD, TTN, TNPO3, etc. (blue nodes represent the microRNAs and the green squares represent the genes). (**B**) Graphical representation of miRNA–target interactions using TFmiR2 and Cytoscape. Blue nodes represent the miRNA, Red nodes and Green nodes represent the target transcription factors.

We identified miRNA–mRNA target interactions through using DIANA mirPath v3.0 (http://snf-515788.vm.okeanos.grnet.gr/) and the networks were visualized by Cytsocape. In this network, we investigated the association between dysregulated microRNAs and muscle protein-encoding genes, hence, this association will reveal the effect of microRNAs dysregulation in the pathogenesis of muscular dystrophy. Interestingly, DMD gene was found to be regulated by miR-495-3p and miR-369-3p, which are significantly dysregulated in the boy and the carriers. Also, the most up-regulated miRNA species, which is miR-409-3p was found to regulate Titin (TTN) and Delta Sarcoglycan protein (SGCD) (Figure 7A). In addition, miR-494-3p also regulates the expression of DAG1 gene encoding dystroglycan which is the central component of dystrophin–glycoprotein complex, TNPO3 gene encoding Transportin-3 which usually found in its mutated form in LGMD, DNAJB6 gene which also the one of the main causes of LGMD, and miR-494-3p is also regulating TOR1AIP1 or Lamin-associated protein 1B.

Finally, we investigated the interplay between circulating microRNAs and some of transcription factors using TFmiR2 and visualized using Cytoscape. As indicated in the network (Figure 7B), microRNAs were found to regulate some crucial factors in TGF-b signaling network that are known to play a pivotal role in controlling the muscle mass and in the neuromuscular junctions, such as SMAD2/3, TGF-bR2. Also, miR-206 was found to regulate several muscular proteins such as MyoD (myoblast determination protein 1), MYOCD (Myocardin), MYOG (Myogenin), and MYF5 (Myogenic Factor 5).

## Discussion

DMD is a monogenic neuromuscular disorder that causes severe muscle degeneration due to inactivating mutations in the DMD gene that result in C-terminal truncated dystrophin protein, which adversely affects its function and elicit mechanical disruption of the sarcolemma [1]. Consequently, for such membrane disruption, Ca^2+^ influx increases, which aggravates the inflammatory responses and activates the apoptotic system in myocytes and thus, augments the dystrophic status in different musculature, a process that eventually becomes fatal when reaches the pulmonary and the cardiac pathophysiology [3,4].

MicroRNAs (miRNAs) constitute a special class of epigenetic regulators that play crucial roles in regulation of gene expression. Generally, miRNAs are released in the circulation in response to normal cellular turnover or because of pathological conditions [15]. In the context of our study, such release occurs secondarily to muscular atrophy/dystrophy as well as due to onset of fibrosis. The types and levels of the released muscle-specific miRNAs (myomiRs) depend on the degree of muscle degeneration, the duration and progression of the disease and the extent of involvement of systems dysfunction especially the cardiopulmonary musculature and hence they can serve for diagnostic/prognostic purposes and to monitor disease progression and treatment effectiveness [17].

In the present study, NGS was utilized to detect modulation in miRNA levels in the plasma and leukocytes of a DMD patient and manifesting and non-manifesting carriers, as an essential screening step to establish a miRNA-based diagnostic methodology as a non-invasive alternative to current diagnostic methods.

Our data show that miR-409-3p was highly elevated in both the plasma and the leukocytes of the DMD patient, to lesser extent in the symptomatic female carriers, but marginally in the asymptomatic carrier (Supplementary Figure S1). miR-409-3p seems to play a role in the regulation of gene expression of gene expression of some muscle-specific protein-coding genes like myotubularin-related protein 7, myosin light chain kinase, myopalladin, cofilin 2 (muscle variant) (predicted targets). miR-409a-3p also has a binding site on the transcripts of mitochondrial ribosomal protein L35 and transforming growth factor-β receptor III genes.

Interestingly, one of the studies that targeted age-associated alterations in skeletal muscles investigated the miRNA expression levels in the aged skeletal muscles of rhesus monkeys. The study reported that the level of miR-409-3p was elevated more than four-folds in the aged muscle cells and hence they linked the miRNA dysregulation with the functional disability and altered metabolism and the degeneration of the muscles accompanied to inflammation [36]. Moreover, the level of miR-409-3p were significantly increased in the exosomes purified from the CSF of Parkinson’s disease patients and it was showed to play a role in neurotrophin signaling pathway [37]. Similarly, miR-409-3p was also reported to be significantly increased in the serum of meningioma patients [38] and it was also up-regulated in other pathological conditions like medulloblastoma [39], glioma [40], traumatic subarachnoid hemorrhage [41], encephalomyelitis [42], and lacunar stroke patients [43]. In a study by Aksu-Menges et al., they identified common miRNA signatures associated with mitochondrial damage in different muscular dystrophies [44]. Six miRNA species including miR-409-3p were found to be dysregulated and associated with mitochondrial damage in different MD groups, therefore contributing to the pathophysiology [44].

Additionally, studies that employed NGS for quantification of the dysregulated miRNAs in the leukocytes of untreated relapsing-remitting multiple sclerosis (MS) patients showed that miR-409-3p and miR-494 significantly exhibited high levels as compared with that of the healthy subjects.

On the other hand, a previous study carried by Meyer et al. [45] identified seven miRNA species that shown to be down-regulated within the process of myoblast differentiation; miR-409-3p was among those decreased species. The contradiction of data reported by that study with our results could be attributed to the fact that the process of myoblast differentiation occurs after muscle injury to promote muscle regeneration, while, in DMD and severe muscle damage, the process of muscle degeneration and regeneration ceases following muscle necrosis and fibrosis, and this may account for the relative increase in the circulating levels of miR-409-3p in the DMD patient and carriers.

Since DMD mutation is located on the X chromosome, females can be carriers for the mutation, without the onset of obvious symptoms. On the other hand, some of the carriers may exhibit muscle weakness and cardiac problems and are usually classified as manifesting carriers. Our hypothesis relies on the variations in miRNA profiles between the two sisters and their mothers. Such differences could, at least in part, account for the occurrence of skeletal muscle weaknesses and that the extent of severity could also be attributed to the differences observed in circulating/cellular levels of dysregulated miRNAs between the two sisters.

Dysregulation of miRNAs profiles could be, at least in part, attributed to the decreased muscular functional performance due to the overall weakness of muscular tissue and thus, they could play significant roles in the onset and development of symptoms besides the presence of faulty dystrophin protein. To highlight such miRNA species, we identified a list of microRNAs that were dysregulated in the symptomatic carriers (the two sisters) but not in their mother (asymptomatic). From such species, miR-494-3p and miR-410-3p were significantly up-regulated in sera and/or cellular fractions of manifesting carriers and in DMD patient.

Comparable and consistent with our data that showed sharp increase in miR-494-3p levels in plasma and in leukocytes of the manifesting female carriers, this miRNA species was also found to be significantly elevated in temporal lobe epilepsy patients [46]. Similarly, circulating miR-1-3p was also up-regulated in the manifesting carriers and it was demonstrated to be associated with Parkinson’s disease [47]. The increase in such miRNA species and their involvement in a wide variety of neurological disorders highlight the potential of these miRNAs as key regulators of neurological circuitry.

In their study, Yamamoto et al. [48] reported that the expression of miR-494-3p was significantly decreased in C2C12 murine myoblast cell line during myogenic differentiation, a condition that is not present in DMD patient and manifesting carriers. Furthermore, the present study suggested that miR-494 negatively regulates mitochondrial biogenesis by down-regulating mitochondrial transcription factor A (mtTFA) and Forkhead Box j3 (Foxj3) during myocyte differentiation and skeletal muscle adaptation to physical exercise. Indeed, mitochondrial dysfunction is one of the main pathological characteristics of DMD since the calcium ions overload causes mitochondrial swelling and the production of reactive oxygen species which consequently lead to pore opening in dystrophin-deficient muscles. The levels of mitochondrial miRNAs could vary depending on the status of the cell and its metabolic demands to regulate the expression of the mitochondrial genome. Lemecha et al. also reported that the expression of miR-494-3p was decreased during skeletal muscle differentiation and related with mitochondrial biogenesis in muscle differentiation and adaptation to exercise in skeletal muscle [49].

Likewise, the levels of miR-376a-3p were highly increased in the carriers, and a similar elevation was also reported in the muscles of myotonic dystrophy patients, which may account for the elevated levels of this species in symptomatic carriers.

miR-410-3p was another member that increased in the manifesting carriers. Clark et al. [50] showed that inhibiting miR-410-3p in stressed cardiomyocytes attenuated the hypertrophic response and the formation of the extracellular matrix. The high CK levels observed in the DMD patient and his symptomatic sisters along with elevated levels of miR-410-3p could be explained in the light of the study of Clark et al.

In contrast with up-regulated miRNAs, miR-144-3p was found to be down-regulated in the three participated female carriers. Such decrease in the circulating levels was also reported in sarcopenia patients [51] who suffered from progressive loss of skeletal muscles. miR-101-3p was also down-regulated and was previously reported to exhibit similar pattern in neuromyelitis optica [52] and familial dysautonomia patients [53]. Moreover, miR-101-3p was also found to be down-regulated in the upper airway skeletal muscles of patients suffering from obstructive sleep apnea hypopnea syndrome [54].

Interestingly, our data revealed that some microRNAs are exclusively increased in the younger sister who suffers from neurological abnormalities and thus, such microRNAs are likely to be involved in brain function and have potential clinical inference and contributing to the neuro-pathophysiology of DMD. Such microRNAs were associated with some neurological disorders, for instance miR-539-5p was up-regulated in serum of this particular carrier and was previously reported to be associated with the onset of epilepsy in all patients’ age categories [55]. Zacharewicz et al. revealed that miR-539 were regulated with age and exercise and were predicted to influence Akt-mTOR signaling, and therefore controlling skeletal muscle mass [56].

Again, circulating miR-410-3p was uniquely increased in serum of the younger sister and this elevated level could be explained based on a relatively higher rate of myocytes turnover.

Circulating miRNAs (plasma levels) may reflect cellular contents, normal cellular turnover, or may indicate accelerated cellular necrosis as a consequence of pathological condition, as is the case of DMD patient or symptomatic carriers. In our study, both DMD patient (DF-1) and sister-1 (DF-3), but not sister-2 (DF-4), exhibited lower circulating levels in plasma as compared with that present in blood cells. Such an increase in circulating level could be attributed to the accelerated myocyte turnover observed in DF-4 (severely symptomatic) but not in DF-3 (milder condition). In the meantime, such an increase could not be observed in DF-1, as the time of sampling coincided with a late disease stage of the patient, at which an anergic response may be operating and shut down ineffective responses such as that regulating miR-410-3p expression.

Additionally, miR-199b-5p significantly decreased only in the leukocytes of the younger sister and this miRNA is known to be down-regulated in the brain tissue retrieved from epilepsy [57] and glioblastoma patients [58]. Similarly, miR-340-3p was also significantly decreased and this species was reported to be also dysregulated in a cell line model of Parkinson’s disease [59].

Also, data analysis showed that some microRNAs are detected in the serum of this female carrier only and could possibly play a crucial role in the brain involvement observed in this girl. Among these miRNAs, miR-206 showed the highest up-regulation levels (Log Fold Change = 8) and is expressed exclusively in this carrier. Serum miR-206 was proposed previously for detecting muscular dystrophy carriers [20] and to have a therapeutic potential. MiR-206 levels was also up-regulated in other neurological diseases like ALS patients [60], myotonic dystrophy patients [61], and glioma [62]. Trifunov et al. analyzed the expression levels of miR-206 in DMD patients and the study discovered that miR-206 can distinguish the DMD and BMD phenotype and can be used to monitor the therapeutic efficacy of corticosteroids treatment [63]. Moreover, similar results were obtained in other studies investigating DMD patients [64] and in dystrophin-deficient animal models [65].

The same carrier also exhibited marked up-regulation of miR-133a-3p (Log Fold Change = 6) and the up-regulation of miR-133a-3p was reported in studies of DMD [66] and myotonic dystrophy [61,67]. Such association with the aforementioned diseases suggests that these species: miR-206, miR-133a-3p, and miR-410-3p are implicated in the neurological impairment and could play roles in the onset of neurological complications observed in the severely manifesting carrier.

In conclusion, the data provided in our study provided an illustrative dissection of miRNA profiles in DMD patients and asymptomatic and symptomatic carriers, which would provide helpful insights to studies concerned with monitoring disease progression of DMD patients as well as identifying female carriers and predicting muscular/neurological involvement in such carriers. In the meantime, our study highlighted miR-409-3p along with other miRNA panel that could serve in diagnostic/prognostic purposes for DMD patients and more importantly for the detection of female carriers which will facilitate accurate genetic counseling. The data analysis revealed that some miRNAs are solely expressed in the DMD patient (as particularly can be seen in blood cells), with the closest member was the severely neurologically affected younger sibling. On the other hand, miRNA profiles in the asymptomatic carrier (the mother; DF-2) and the slightly symptomatic carrier (the elder sister; DF-3) exhibited comparable trends, especially in the blood cell samples and such relationship has been highlighted in the discussion. Future studies should be carried out for validating the correlation of the miRNA with the dystrophic status of the muscles through using a primary muscle culture or DMD animal model (mdx mice or GRMD dog model) and to test the therapeutic potential of selected miRNA species.

## Supplementary Material

Supplementary Figure S1

Supplementary Tables S1-S11

## Data Availability

The datasets used and/or analyzed during the current study are available from the corresponding author on reasonable request.

## Abbreviations

CK: creatine kinase
DEM: differentially expressed miRNA
DMD: Duchenne muscular dystrophy
GRMD: golden retriever muscular dystrophy
MLPA: multiplex ligation-dependent probe amplification
MS: multiple sclerosis
NGS: next-generation sequencing
