# Supplementary material for: miRNome profiling in Duchenne muscular dystrophy; identification of asymptomatic and manifesting female carriers
Source: Biosci Rep. 2021 Sep 17;41(9):BSR20211325. doi: 10.1042/BSR20211325 (PMC8450315; doi:10.1042/BSR20211325)
Supplement: Supplementary Figure S1 [file BSR-2021-1325_supp.pdf]

List of Supplementary Figure Legends:

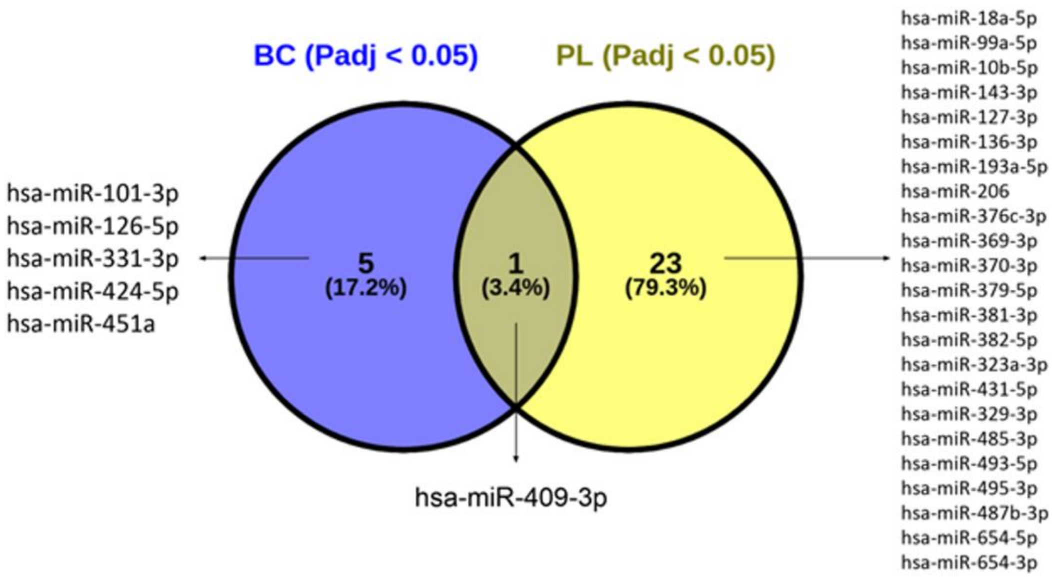

Supplementary Figure 1: **Common and exclusive microRNAs in the plasma and the blood cells**; Venn Diagram showing the plasma-specific microRNAs, blood cells-specific microRNAs and the common dysregulated species present in the plasma as well as blood cells of the family members. BC=Blood cells and PL=plasma. Venn Diagram was generated based on stringent p-value (adjusted p-value < 0.05).
