# Supplementary material for: miRNome profiling in Duchenne muscular dystrophy; identification of asymptomatic and manifesting female carriers
Source: Biosci Rep. 2021 Sep 17;41(9):BSR20211325. doi: 10.1042/BSR20211325 (PMC8450315; doi:10.1042/BSR20211325)
Supplement: Supplementary Tables S1-S11 [file BSR-2021-1325_supp1.zip › BSR-2021-1325_suppST1.docx]

| **Primer** | **Primer sequence** |
| --- | --- |
| cel-miR-39-3p (spike in control) | 5’- tcaccgggtgtaaatcagcttg -3’ |
| hsa-miR-409-3p | 5’- gaatgttgctcggtgaacccct -3’ |
| hsa-miR-410-3p | 5’- aatataacacagatggcctgt -3’ |
| hsa-miR-494-3p | 5’- tgaaacatacacgggaaacctc -3’ |
| hsa-miR-206 | 5’- tggaatgtaaggaagtgtgtgg -3’ |
| hsa-miR-624-3p | 5’- cacaaggtattggtattacct -3’ |
| hsa-Let-7a-3p | 5’- ctatacaatctactgtctttc -3’ |
| hsa-miR-424-5p | 5’- cagcagcaattcatgttttgaa -3’ |
| hsa-miR-144-3p | 5’- tacagtatagatgatgtact -3’ |
